# A prostate derived commensal *Staphylococcus epidermidis* strain prevents and ameliorates induction of chronic prostatitis by UPEC infection

**DOI:** 10.1038/s41598-018-35818-1

**Published:** 2018-11-27

**Authors:** Stephen F. Murphy, Christel Hall, Joseph D. Done, Anthony J. Schaeffer, Praveen Thumbikat

**Affiliations:** Department of Urology, Feinberg School of Medicine, Northwestern University, Chicago, IL 60611 United States

## Abstract

Chronic prostatitis/Chronic pelvic pain syndrome (CP/CPPS) is a common syndrome with limited therapies and an unknown etiology. Previously, our laboratory has defined a potential role for pathogenic infection in disease onset. Intra-urethral infection with a uropathogenic *Escherichia coli* strain isolated from a CP/CPPS patient, CP1, induces prostatic inflammation and tactile allodynia in mice. We have also demonstrated that a prostate specific *Staphylococcus epidermidis* bacterial isolate, NPI (non-pain inducing), from a healthy subject reduces pain and inflammation in an experimental autoimmune prostatitis (EAP) murine model. Here we focus on the interplay between these human isolates in the context of prostatitis development and resolution. NOD/ShiLtJ mice were inoculated with either NP1 or CP1, or combinations of both. Infection with CP1 induced pelvic tactile allodynia after 7 days, while NPI instillation alone induced no such response. Instillation with NPI 7 days following CP1 infection resolved pelvic tactile allodynia and prophylactic instillation 7 days prior to CPI infection prevented its onset. Prophylactic NPI instillation also prevented CP1 colonization of both prostate and bladder tissues. *In vitro* analyses revealed that CP1 and NPI do not directly inhibit the growth or invasive potential of one another. Immunological analyses revealed that specific markers associated with CP1-induced pelvic allodynia were decreased upon NPI treatment or repressed by prophylactic colonization. This study demonstrates that a commensal bacterial isolate can inhibit the colonization, pain responses, and immunological activation to uropathogenic bacteria, emphasizing the power of a healthy prostatic microflora in controlling health and disease.

## Introduction

Prostatitis is a common medical complaint among men of all ages, accounting for over 2 million urology clinic visits each year in the U.S. alone^1^. It is subcategorized into four classifications of which Chronic Prostatitis/Chronic Pelvic Pain Syndrome (CP/CPPS) is the most common, accounting for more that 90% of all diagnoses^2,3^. At present little is known about the etiology of the syndrome and thus effective treatment options are limited. It is clinically thought to be non-infectious but emerging evidence suggests a potential role for bacteria in its development. Symptomatically, CP/CPPS is manifested as chronic pain in the pelvic and genital regions. Additional symptoms such as lower urinary tract dysfunction and decreased quality of life are also frequently reported, all with varying degrees of severity^4,5^.

CP1 is a uropathogenic *Escherichia coli* (UPEC) strain that was isolated by our laboratory from the prostate of a patient with CP/CPPS^6^. CP1 has been characterized, sequenced and phylogenetically compared to other UPEC strains and has been shown to be a B2 strain with virulence characteristics that perhaps suit adaptation to the prostate^7^. CP1 can drive prostatitis development in NOD/ShiLtJ and C57BL/6 mice, but only NOD/ShiLtJ mice develop pelvic tactile allodynia^6,8^. Immunologically, the tactile allodynia response has been shown to be driven by Th1 and Th17 meditated inflammation of the prostate and is maintained even after clearance of the bacteria^8^.

Unlike CP1, the Non Pain-Inducing (NPI) *Staphylococcus epidermidis* strain was isolated from the prostate of a healthy human subject^9^. *S*. *epidermidis* is a common skin commensal and has been demonstrated to be capable of regulating responses to invading pathogens, such as *S*. *aureus*^10–12^. Using the prostate-derived *S*. *epidermidis* we have previously shown that this strain can efficiently colonize mouse prostate and bladder tissues^9^ and does not induce prostate inflammation or pelvic tactile allodynia in either NOD or C57BL/6 mice^9^. We have also demonstrated that this NPI strain is distinct from another *S*. *epidermidis* strain (7244) isolated from CP/CPPS patient prostate secretions^13^, specifically in the ability to induce and modulate pain responses. We are currently investigating the phylogenetic differences between them and which clades these strains belong to. In our xenogeneic experimental autoimmune prostatitis (EAP) murine model of CP/CPPS^14^, we have shown that NPI has the capacity to ameliorate EAP-induced pelvic tactile allodynia and immune activation, via increased expression of the negative regulators of the adaptive immune response, PDL1 and PDL2^9^.

In this study we examine the interplay of a prostate-derived commensal and a pathogen in the initiation and maintenance of CP/CPPS. Using NOD mice we demonstrate that NPI instillation during an ongoing CP1 infection can reduce CP1-induced tactile allodynia. Prophylactic instillation with this commensal strain reduces CP1 prostate colonization and attenuates tactile allodynia responses. Analysis of strain-strain interactions reveal that these *in vivo* findings are likely due to altered host immune responses to the bacterial strains rather than due to direct bacterial competition. Taken together this study identifies the potential roles of prostate specific bacterial strains to be either harmful or beneficial to male urological health.

## Materials and Methods

### Animal Use

Male 6–8 week old NOD/ShiLtJ (NOD) mice were procured from Jackson Laboratory (Bar Harbor, ME). Animal procedures and experiments were all performed according to the protocol approved by the Institutional Animal Care and Use Committee (IACUC) at Northwestern University. The university has an assurance on file (A3283-01) at the Office of Laboratory Animal Welfare. Protocol reviews are conducted according to the regulations set out by the United States Public Health Service (USPHS) and applicable laws. IACUC composition meets all requirements of policies from the USPHS and Animal Welfare Act Regulations. All procedures and experiments performed here adhere to these regulations. Experimental mice were randomized between cages at experimental start point. Blinding was performed for all experiments and subsequent analyses. Control animals were uninfected/untreated naïve mice on which matching behavioral testing and post-experiment analyses were performed.

### Human Bacterial Sample Collection

Patient bacterial sample collection was performed in accordance with the approved protocol STU00046489 from the Institutional Review Board (IRB) at Northwestern University and Memorial Hospital. A patient waiver of consent for post-collection sample analyses was approved under section 45CFR46.116d(1–4). Bacterial samples were deemed to be prostate localized if bacterial counts were 1-log fold greater in expressed prostatic secretion (EPS) or voided bladder 3 (VB3) samples compared to VB1 and VB2 samples. The Meares-Stamey 4-glass test was used to collect patient samples in the clinic. Bacterial identification was performed in the clinical microbiology using the automated BD platform based on bacterial strain susceptibility, growth and sensitivity to specific antibiotics and conditions.

### Intra-urethral Bacterial Infection

As described in previous publications^6,8^, the bacterial strains used for infection were CP1, a Group B Uropathogenic *Escherichia coli* (UPEC) strain and Non-Pain Inducing (NPI), a *Staphylococcus epidermidis* strain isolated from a healthy control subject and thus deemed a commensal. Briefly, both strains were grown in Luria Broth (LB) overnight, with shaking, at 37 °C followed by a second overnight static subculture. Cultures were spun down and concentrated in sterile Phosphate-buffered Saline (PBS) (Thermofisher) to an OD420 value of 1 ± 0.01, resulting in bacterial numbers of 2 × 10^10^ per ml. 10 μl or 2 × 10^8^ bacteria were infection/instilled by intra-urethral catheter injection into mice anesthetized by isoflurane.

### Von Frey Behavioral Testing

As described previously, tactile allodynia as referred visceral hyperalgesia was measured using Von Frey behavioral testing. Briefly, following can acclimating period of one hour in the testing room, mice were placed into a Plexiglas chamber with a wire grid floor and allowed to acclimate for 2–3 minutes. Von Frey filaments of varying forces (0.04, 0.16, 0.4, 1 and 4 g) (Stoelting)^15^ were applied 10-times to suprapublic regions for 1–2 seconds at 5 second intervals. Responses were measured as positive when mice displayed behaviors such as retraction of the abdomen, jumping or immediate licking and scratching of the application site. Testing was performed in the same room by the same investigator at the same time at days 0, 7 and 14, post-infection/instillation. Responses recorded at day 0 were used as the baseline score prior to treatment. Scores are displayed as percentage response increase/decrease from baseline.

### Tissue Colony Formation Assay

At experiment endpoint, Day 14, prostate and bladder tissues were surgically removed under sterile conditions. Both tissues were minced using scissors and dissociated by addition of a filtered solution of 1 mg/ml collagenase D (Roche), 10 mM HEPES (Mediatech), and 0.01% DNase I (Sigma) in RPMI 1640 (Mediatech) with shaking at 37 °C for 2 hours. Following this tissues were filtered through a 40 μm nylon filter and the suspension plated on LB agar, Eosin methylene blue (EMB) agar (specific for gram-negative bacteria) or Columbia Naladixic Acid with 5% Sheep’s Blood (CNA) agar (specific for gram-positive strains). Plates were incubated overnight in a 37 °C incubator.

### Deferred Growth Inhibition Assay

Bacterial growth inhibition assay was performed as described^16^. Briefly, a vaporizer bottle was cleaned and sterilized under UV light and then rinsed with 70% ethanol including through the spray mechanism. Immediately prior to use the bottle was rinsed with sterile LB to ensure that the inoculum was not affected by the presence of ethanol. A single colony overnight culture of both strains was prepared by streaking of glycerol stocks onto a dry sterile LB agar plate and incubation at 37 °C. The following day single colonies were picked and 10 ml of sterile LB inoculated and returned to the incubator at 37 °C overnight. For the central dot of bacteria 25 ul of this overnight culture was pipetted onto a dry sterile LB Agar plate, allowed to dry at room temperature to limit culture spread and incubated overnight at 37 °C. The spray inoculum was prepared by dilution of the overnight culture 10-fold into sterile LB broth. An excess of 250 ul per plate was prepared, which equates to approximately 3-spray pumps. Strains were co-cultured by spraying the diluted culture onto the dotted-agar plate at a distance of 15 cm. This plate was returned to the incubator and grown overnight at 37 °C. The following day the distance between the central colony and the bacterial spray lawn was measured.

### Gentamicin Protection Competition Assay

The CP1 and NPI bacterial strains were grown and concentrated as described above. The normal human prostate epithelial cell line RWPE-1 (ATCC® CRL-11609™) in K-SFM media with supplements (Gibco, 17005-042) was seeded at 2.5 × 10^5^ cells per well of a 6-well tissue culture plate and grown overnight at 37 °C. Bacterial strains at a multiplicity of infection (MOI) of 10 were then added to the cells. Two inter-strain comparisons were performed. 1. Adherence -vs- Invasion: the primary strain was incubated for 2 hours at 37 °C and 5% CO_2_ followed by 4 washes in sterile PBS (Thermofisher), following which media containing 50 ug/ml of gentamicin was added to the cells and the plate returned to the incubator for 30 mins. Cells were rewashed 4 times with sterile PBS and the secondary strain added and incubated for 2 hours at 37 °C and 5% CO_2_. 2. Invasion -vs- Invasion: A 2 hour incubation with the primary strain followed by gentamicin treatment as above, followed by the same incubation time and treatment, including gentamicin, with the secondary strain. Following these incubations, cells were lysed using 0.5% Trypsin (Gibco) containing 0.1% Triton-X (Thermofisher), serially diluted and plated on EMB or CNA Agar plates. Plates were incubated overnight at 37 °C, colonies counted and colony forming units calculated.

### Luminex Chemokine Cytokine Bead Array

At experimental endpoint, Day 14, prostate tissues were surgically removed and digested as described above. Single cell suspensions of the iliac lymph nodes were prepared by manual mincing and dissociation of the node through a 40um nylon mesh using sterile PBS. These samples were then added to a Milliplex® MAP kit plate and the assay performed according to manufacturers instructions. Protein content of chemo/cytokines was normalized against total protein content, assessed by Bicinchonic Acid Assay (BCA) (Pierce) according the manufacturers instructions.

### Statistical analyses

All statistical analyses and tests were performed using data collected and collated in Microsoft Excel™, data was then analyzed using Graph Pad Prism™. Specific tests used, number of technical and biological replicates, and murine *n* values are indicated in figure legends. The *t*-tests performed were unpaired Student’s *t-test* and two-tailed, one-way Analysis of Variance (ANOVA) tests included post-hoc multi-comparison Tukey analyses. Data are depicted as mean ± standard error of the mean (S.E.M) unless otherwise sated, with *P* < 0.05 signifying statistical significance. *P < 0.05, **P < 0.01, ***P < 0.001, ****P < 0.0001.

## Results

### NPI instillation significantly reduces tactile allodynia in CP1-infected mice

**Figure 1:**
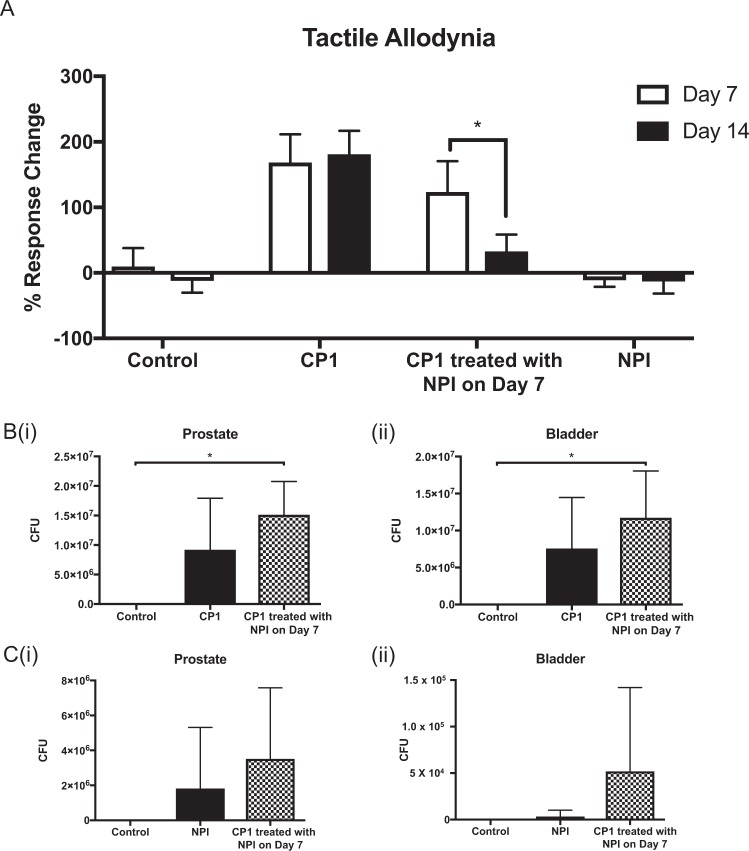
NPI instillation ameliorates CP1 infection-induced pelvic tactile allodynia. (**A**) Pelvic tactile allodynia responses displayed as percent response change above baseline for each animal at Day 7 and 14 post-treatment. (**B**) Tissue colony counts for (i) prostate and (ii) bladder tissues displayed as CFU from EMB, gram-negative specific agar plates. (**C**) Tissue colony counts for (i) prostate and (ii) bladder tissues displayed as CFU from CAN with 5% sheep’s blood agar plates, specific for gram-negative bacteria. Combined data of N = 2 experiments, with n = 4/5 mice per group. Control animals are un-treated naive mice. Statistical tests performed: One-way ANOVA with post-hoc Tukey’s multiple comparison test between groups. *p < = 0.05.

The NPI bacterial strain is capable of ameliorating tactile allodynia and prostatic inflammation in an EAP model of CP/CPPS^9^. We sought to assess whether NPI could perform a similar therapeutic function in the CP1-induced model of prostatitis. NOD mice were infected with CP1 and prostatitis was allowed to develop for 7 days, following which one group of infected mice was intra-urethrally treated with NPI instillation. Figure 1A depicts the tactile allodynia response change to bacterial instillation/infection at 7 and 14 days post-treatment. CP1 infection alone induces an increase in Von Frey behavioral response at both time-points. In the dual bacterial treated group, CP1 induced tactile allodynia responses at day 7 that were significantly decreased at day 14, 7 days post-instillation with NPI. To investigate whether this decrease was due to alterations in CP1 colonization upon NPI instillation we performed tissue colony formation assays using selective growth media on both bladder and prostate tissues from infected mice. Figure 1B(i & ii) show that NPI instillation does not decrease the tissue colony counts of the CP1 isolate compared to that observed in CP1-infected mice alone. Furthermore Fig. 1C(i & ii) demonstrate that prior infection with CP1 does not diminish the ability of NPI to colonize the prostate or bladder of NOD mice compared to NPI instillation alone. Taken together these data demonstrate that NPI instillation into CP1-infected mice significantly reduces tactile allodynia without decreasing CP1 colonization, suggesting alternative mechanisms may mediate these effects.

### Prophylactic NPI instillation decreases CP-1 colonization and CP1-induced tactile allodynia

**Figure 2:**
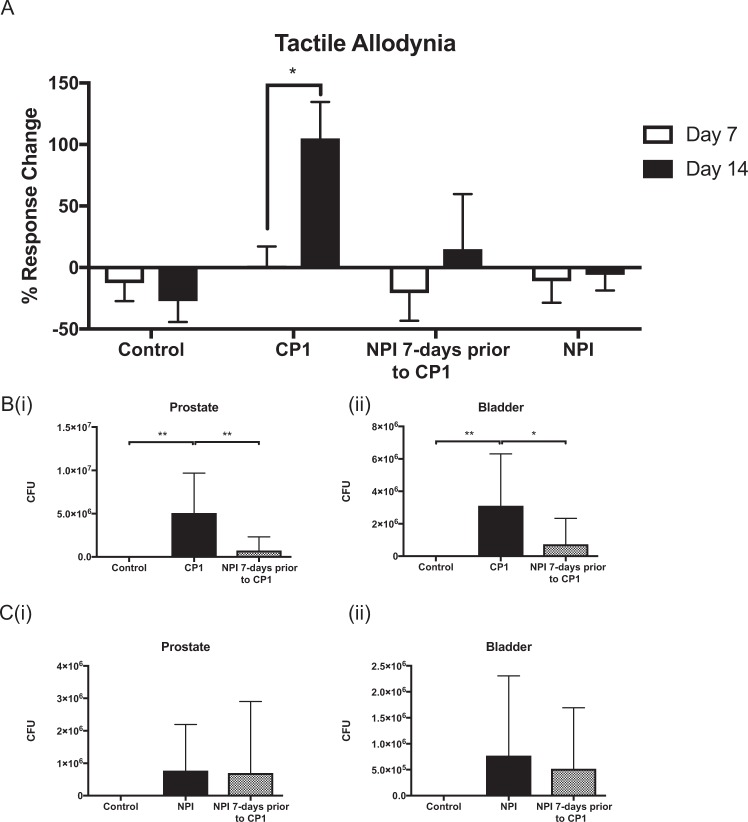
NPI prophylactic instillation prevents development of CP1 induced pelvic tactile allodynia. (**A**) Pelvic tactile allodynia responses displayed as percent response change above baseline for each animal at Day 7 and 14 post-treatment. (**B**) Tissue colony counts for (i) prostate and (ii) bladder tissues displayed as CFU from EMB, gram-negative specific agar plates. (**C**) Tissue colony counts for (i) prostate and (ii) bladder tissues displayed as CFU from CAN with 5% sheep’s blood agar plates, specific for gram-negative bacteria. Combined data of N = 2 experiments, with n = 4/5 mice per group. Control animals are un-treated naive mice. Statistical tests performed: One-way ANOVA with post-hoc Tukey’s multiple comparison test between groups. *p < = 0.05, **p < = 0.01.

Given that the NPI isolate was obtained from a healthy prostate and was hypothesized to be commensal in nature, we next wanted to examine the impact of prophylactic instillation and colonization by NPI on CP1-induced prostatitis development. To do so we performed a second dual bacterial treatment experiment. NPI was instilled into mice and 7 days later these mice were infected with CP1. Figure 2A depicts the Von Frey behavioral responses to these treatments at both day 7 and day 14 post-instillation. As previously demonstrated CP1 infection alone significantly increases tactile allodynia responses above control while NPI instillation does not. In the dual bacterial treated group we observed that NPI prophylactic instillation abrogates the ability of CP1 to induce tactile allodynia in NOD mice. To examine the effect of NPI on the colonization of CP1 we performed tissue colony formation assays on both prostate and bladder tissues. Figure 2B(i & ii) show that NPI instillation significantly reduces colony counts of CP1 compared to CP1 infection alone. In addition, secondary infection with CP1 in NPI-instilled animals did not decrease colony formation, Fig. 2C(i & ii). Taken together these data demonstrate that prophylactic instillation with NPI abrogates the colonization and tactile allodynia responses to CP1 in NOD animals. As opposed to the findings observed with NPI treatment, prophylactic effects of NPI may be a result of decreased colonization by CP1 in these animals.

### NPI and CP1 bacterial strains do not significantly inhibit the growth or invasive potential of one another

**Figure 3:**
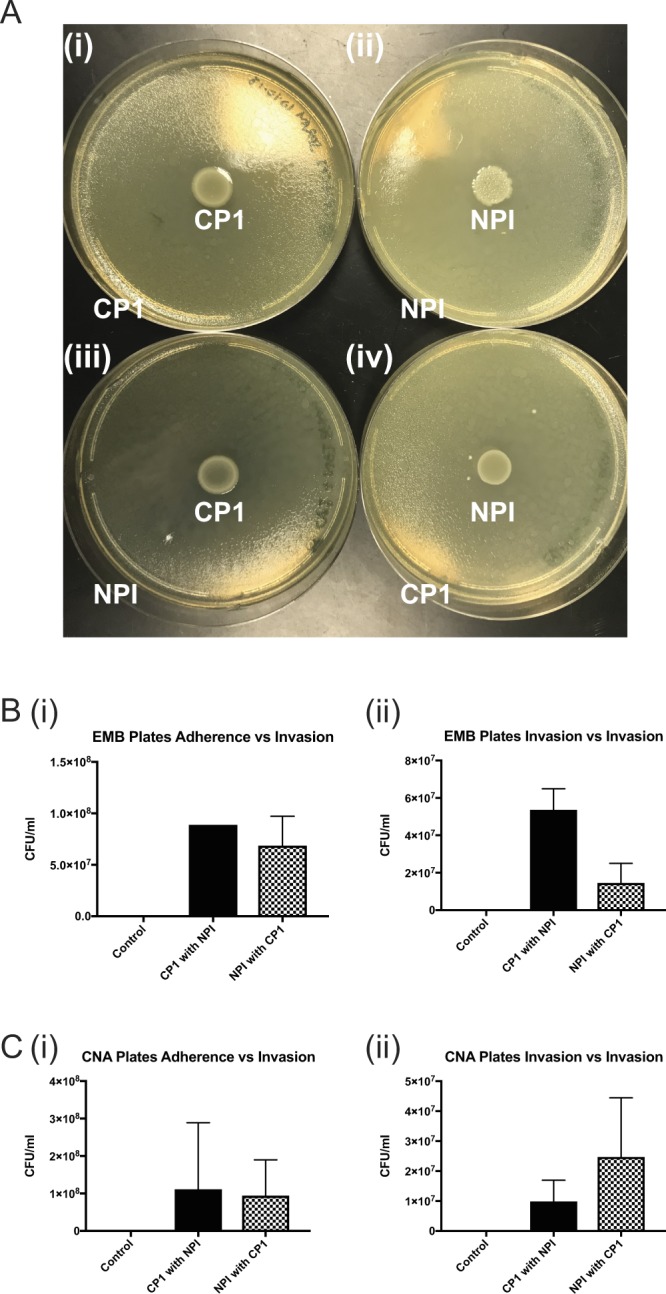
CP1 and NPI do not directly inhibit the growth or invasive potential of one another. (**A**) Deferred growth inhibition assay: (i) Central inoculum CP1 with spray/lawn of CP1. (ii) Central inoculum of NPI with spray/lawn of NPI. (iii) Central inoculum of CP1 with spray/lawn of NPI. (iv) Central inoculum of NPI with spray/lawn of CP1. N = 3, representative images of one experiment shown. (**B**) Colony counts from the adapted gentamicin protection assay comparing (i) Adherence versus Invasion and (ii) Invasion versus Invasion of CP1 and NPI from EMB agar, selective for gram-negative bacteria. (**C**) Colony counts from the adapted gentamicin protection assay comparing (i) Adherence versus Invasion and (ii) Invasion versus Invasion of NPI and CP1 from CNA with 5% sheep’s blood agar, selective for gram-positive bacteria. N = 2, combined data displayed. Statistical tests performed: One-way ANOVA with post-hoc Tukey’s multiple comparison test between groups.

As the prophylactic treatment with NPI demonstrated that NPI has the potential to directly inhibit CP1 colonization we next wanted to assess strain - strain interactions between the bacterial isolates. An *in vitro* deferred growth inhibition assay, Fig. 3A(i–iv), was performed and demonstrated that neither CP1 nor NPI directly inhibits the growth of the alternate isolate^16^. This was shown by the lack of inhibited growth between the central colony and the bacterial lawn of the opposite strain, Fig. 3A(iii & iv), with both growing equally well as their control “same-strain” counterparts, Fig. 3A(i & ii). Images of relevant individual control plates for these of these growth conditions are shown in Supp. Fig. 1(i–v). To examine this further we used an adapted form of the gentamicin protection assay to examine the interaction of these microbes in terms of their ability to adhere and invade prostate epithelial cells. Figure 3B shows colony counts from EMB plates, specific to CP1, from lysed RWPE-1 prostate cells that were infected with CP1 followed by NPI or vice versa. Longer incubation times in 50 ug/ml gentamicin allowed differentiation between bacterial adherence and invasion and the ability of one isolate to impact the potential of the other assessed by the order of infection. No significant differences in colony counts were observed for CP1, Fig. 3B(i & ii) or for NPI, Fig. 3C(i & ii) regardless of the order of infection for either adherence or invasion of the alternate isolate. These data therefore suggest that the differences in mouse responses to combinations of bacteria are more likely to be host derived than to emerge from any direct microbial interactions.

### NPI alters CP1 infection-induced prostate immune responses

**Figure 4:**
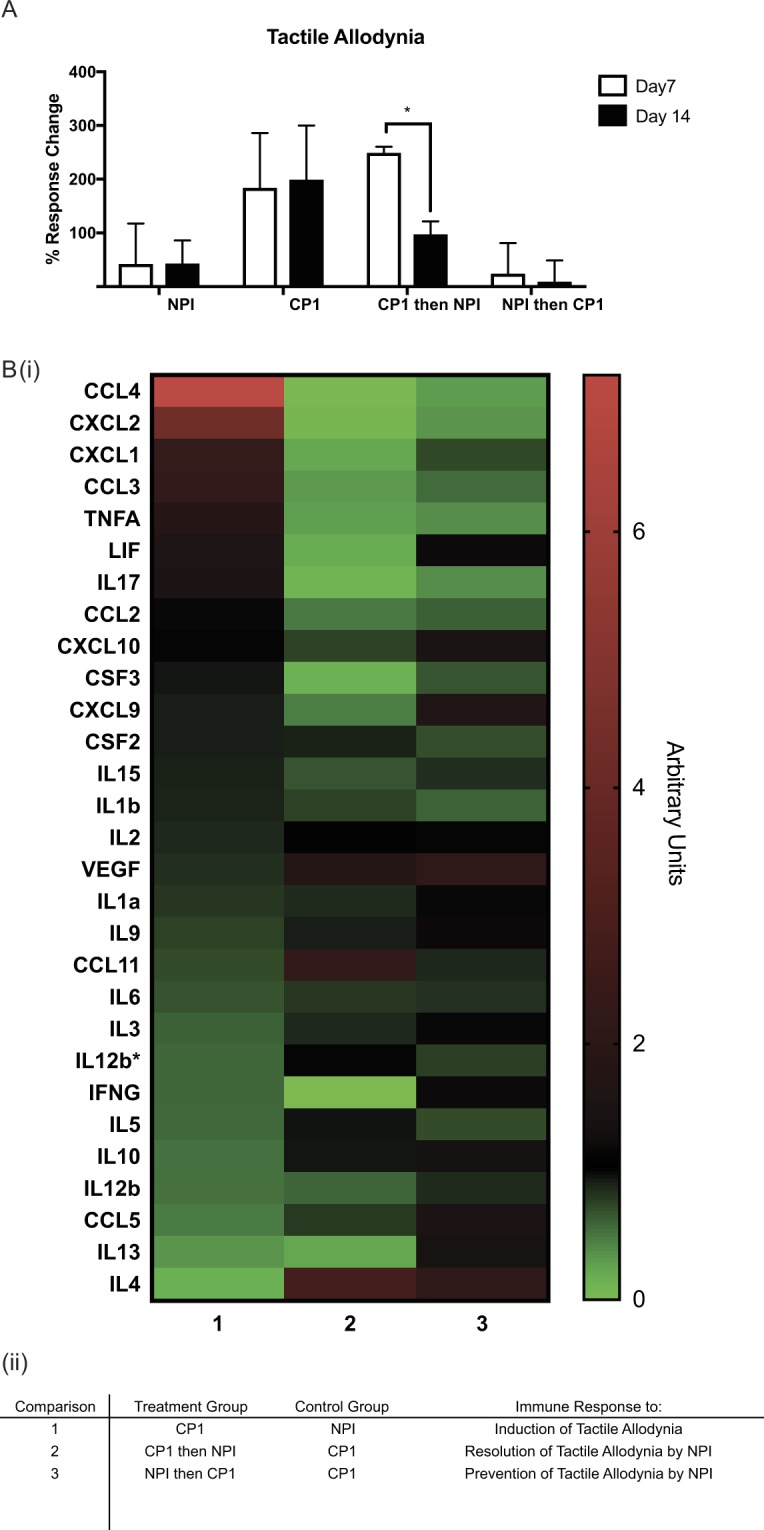
NPI alters CP1 infection-induced prostate immune responses. (**A**) Pelvic tactile allodynia responses displayed as percent response change above baseline for each mouse group at Day 7 and Day 14 post-treatment. N = 1, with n = 3/4 mice per group. Statistical tests performed: One-way ANOVA with post-hoc Tukey’s multiple comparison test between groups, p < = 0.05. (**B**) Luminex chemokine and cytokine bead array data from prostate lysates displayed as (i) heat-map of five comparisons between mouse groups. (ii) Tabular breakdown of comparisons performed. N = 1, with n = 3/4 mice per group.

The data presented above demonstrate that although NPI alters CP1-induced mice tactile allodynia responses and colonization, we did not observe direct interactions between isolates *in vitro*. In order to address this apparent disparity we sought to understand the immunological changes of the host in response to the isolates either alone or in combination. We performed a luminex bead chemokine and cytokine array on tissue lysates from prostates of treated NOD animals. The luminex array determined specific protein levels of 29 analytes and how these were altered between mouse groups. Figure 4A, depicts the tactile allodynia responses from four groups of animals, NPI instillation and CP1 infection alone, CP1-infection with NPI treatment and NPI-instillation with CP1 infection. As previously shown NPI instillation both therapeutically reduces and prophylactically prevents tactile allodynia in NOD animals. Using these animal groups we performed pair-wise analyses of the luminex bead array dataset. Figure 4B(i & ii), depicts these results as a heatmap, each column representing one of these analyses. Analyzing the data in this manner allows for specific comparisons to be made between groups.

The leftmost column shows the protein analytes from CP1-infected animals relative to the NPI-instilled group, i.e. induction of tactile allodynia. These data indicate that the host immune response to the CP1 pathogen include increased expression of CCL3/4, IL17, LIF and CXCL1/2 among others, while simultaneously decreased expression of the Th2 associated cytokines IL4 and IL13. Supp. Fig. 2A is a graphical representation of this analysis alone showing the relative expression of the analytes in the CP1-infected group compared to NPI-instilled (shown as a value of 1). Supp. Fig. 2B,C show a Search Tool for the Retrieval of Interacting Genes/Proteins (STRING) analysis of the proteins, with an increased expression cut-off of >= 1.4, the relationship between these proteins and the five most statistically significant gene ontology (GO) biological process terms associated with the comparison. The most significant of these being “inflammatory response” and “positive regulation of response to external stimulus”, which confirm a host response to a bacterial isolate that is a known pathogen, CP1. Supp. Fig. 2D,E, show similar analyses of the analytes with <= 0.8 fold expression compared to the NPI-instilled group.

The second comparison, column 2, depicted in Fig. 4B is that between CP1-infected mice with NPI treatment versus CP1 infection alone. This analysis reveals the analytes that are altered during resolution of tactile allodynia by NPI. Those increased include IL4, CCL11 and VEGF, while those down-regulated include CCL3/4, IL17 and CXCL1/2. Supp. Fig. 3A, shows this analysis alone with the STRING and GO biological process terms depicted in Supp. Fig. 3B,C (up-regulated >= 1.4) and Supp. Fig. 3D,E (down-regulated <= 0.8). Here we observed increases in analytes associated with terms such as “positive regulation of cell migration” and most notably a significant number of decreased analytes belonged to the terms “response to a molecule of bacterial origin” and “response to lipopolysaccharide”. Comparing the first analysis with the second in Fig. 4B, reveals an observable switching of increased versus decreased analytes between these two groups, indicative of a reversal of immune activation by CP1 upon treatment with NPI.

The third column in Fig. 4B, shows an analysis between mice instilled with NPI and then infected with CP1 compared to CP1 alone, i.e. prevention of tactile allodynia by CP1. Increased analytes include VEGF, IL4 and CCL5 and decreased CCL2/3/4 and IL17. Supp. Fig. 4(A–E) show this analysis alone along with the STRING analyses and GO biological process terms associated with it. Most notably here, we again observed a significant decrease in proteins associated with the “cellular response to lipopolysaccharide”. From Fig. 4B(i), it is also observed that analyses 2 and 3 show similar patterns of protein expression to one another, indicating that NPI may be actively preventing onset of tactile allodynia via the host immune response.

The fourth analysis, Supp. Fig. 5, compares mice instilled with NPI and then infected with CP1 with the NPI-instilled only group, depicting reduced tactile allodynia induction by CP1. Here we see increased expression of CCL4, CXCL1/2 and Lif and that while these analytes are similar to those observed in analysis 1 (induction of tactile allodynia), the increases seen here are comparatively blunted. In addition to analytes such as IL4/5 and IL17 are decreased in this analysis, with IL17 being of particular interest given its high expression in the CP1-infected group. Supp. Figs. 5(B–E) shows respective STRING and GO biological process terms, which include regulation of both adaptive and innate immune responses.

The final pair-wise analysis we performed, Supp. Fig. 6A, is the comparison between CP1-infected mice treated with NPI and NPI-instilled groups, i.e. resolved response to CP1. Here we see increased expression of CCL11 and VEGF with decreases in multiple analytes including IFNG, IL17 and CCL4, along with respective STRING and GO biological process graphs, Supp. Fig. 6(B–E). Of note here is the decrease in expression of proteins associated with the term “positive regulation of cytokine production” indicating that this process is abrogated upon treatment of CP1-infected mice with NPI. Luminex analysis was also performed on lysates from the iliac lymph nodes of these animals, Supp. Fig. 7A(i & ii) & B (i – v). The data here mirrors that seen in the prostate, with a distinct shift between analyte expression levels in analysis 1 (induction of tactile allodynia) compared to analysis 2 and 3 (resolution and prevention of tactile allodynia respectively). The data here is of decreased power owing to a combination of decreased mouse numbers and sample availability. As such it is included in the supplementary figures as supporting information. Taken together these data demonstrate increased expression of a number of chemokines and cytokines in the prostate of mice infected with CP1 that have high tactile allodynia responses, which are reversed upon treatment by NPI instillation. The immune response to NPI prophylactic prevention of CP1-induced tactile allodynia mirrors that observed with therapeutic intervention of NPI, suggesting that NPI is capable of both inducing and maintaining immune responses that protect against pathogen induced inflammation and tactile allodynia.

## Discussion

The functional effects of microflora and specific commensal and pathogenic bacterial strains in the prostate and bladder tissues is not well understood. Our previous studies have focused on two human isolates individually, CP1 as an initiation factor in a murine model of CP/CPPS and NPI as a therapeutic and immuno-modulator in the EAP murine model^6,8,9^. Here we combined these two distinct findings examining both the effect of prophylactic and therapeutic intervention with the NPI commensal bacterial strain in the context of pathogenic infection. The ability of NPI to reduce CP1 induced tactile allodynia without directly impacting pathogen colonization indicates the importance of local host immune activation, and restoration of balance, in mediating CP/CPPS. This is highlighted when we compared the chemokine and cytokine secretion profiles of mice treated with CP1-alone versus NPI-alone, to CP1 with NPI versus CP1 alone (Fig. 4. Analysis 1 compared to Analysis 2). In tandem with significant decreases in tactile allodynia and highlighted visually on the heat map (Fig. 4B (i)) it is clear that there is a distinct switching of expression patterns of multiple analytes.

In the CP1-induced tactile allodynia mice we demonstrate that the C-C Motif Chemokine Ligand (CCL) proteins 3 and 4 are increased compared to the NPI-instilled group. The CCL3/4 proteins and their family members have known roles in mediating pain responses across multiple models^17^. Our lab has shown a role for both CCL2 and CCL3 in mediating EAP induced tactile allodynia^18^ and others have shown that CCL4 is involved in neuropathic pain resulting from sciatic nerve injury as an initiating factor^19^. The C-X-C Motif Chemokine Ligand (CXCL) proteins CXCL1 and 2 are also upregulated by CP1 and CXCL1 has been shown to maintain neuropathic pain and is increased in affected spinal cord tissues^20^, whereas CXCL2 serves as an initiating factor in sciatic nerve injury. CXCL1 and CCL2/4 and 5 have also been shown to mediate neuronal glial communication to modulate nociceptive signal transmissions^21^. More specific to the prostate, CXCR3 is a known mediator of prostate adaptive cell infiltration^22^ and acts a receptor for multiple chemokines including, CXCL9/10 and 11^23^. Thus, increases in the CCL and CXCL chemokines matches well with previously published work on both pain and inflammation. Interestingly, a recent study demonstrated increased expression of CCL1 in men with enlarged prostates and increased expression of CCL11 in men with prostate cancer, a further indication of the role of these chemokine’s in prostate disease states^24^. Another analyte that was increased in CP1 infected mice was leukemia inhibitory factor (LIF). LIF expression is increased locally during skin inflammation and may have anti-inflammatory effects. In the context of pain it has been shown that administration of low doses of LIF locally can reduce inflammatory mechanical hyperalgesia^25^. Finally CP1 infected mice were also shown to have increased levels of IL17, recapitulating our previously published data where we observe increased IL17 expression in CP1-infected mice and mice with EAP^26^.

NPI instillation into CP1-infected mice reversed these increases, suggesting that NPI exerts its effects via modulation of the host response to CP1. Our analyses revealed that CP1 infected animals treated with NPI show reduced levels of CCL3/4, CXCL1/2 and IL17 compared to the CP1 alone group. NPI treatment during an ongoing CP1 infection increased the level of expression of the Th2 cytokine IL4, which was shown to be down-regulated in the CP1-infected group. This indicates a potential mechanism for NPI ameliorating tactile allodynia via skewing towards a Th2/Treg type of adaptive immune response, which would protect against IL17 and CCL3, induced tactile allodynia.

NPI colonization prophylactically, provided significant protection against CP1 pathogen induced tactile allodynia and inflammation. These findings replicate to a significant degree a recent study where researchers examined commensal versus pathogenic colonization in the context of intestinal immune development and disease^27^. Bereswill *et al*. demonstrated that the pathogenic *Campylobacter jejuni* strain was capable of inducing epithelial cell apoptosis upon infection but prophylactic instillation of a commensal *Lactobacillus johnsonii* strain prevented this. Furthermore the authors also show that peroral challenge with *L*. *johnsonii* also attenuated the systemic, intestinal and extra-intestinal proinflammatory cytokine secretion profile associated with *C*. *jejuni* infection^27,28^. The parallels between this study and the data we present are clear, although with some distinct differences, most notably the ability of prophylactic NPI to reduce CP1 colonization. No such colonization differences existed between *C*. *jejuni* and *L*. *johnsonii*. Certain *S*. *epidermidis* strains can act as a facultative pathogens and can form biofilms on surgical implants^29^ so it is possible that NPI protects the colonization niche of CP1 from bacterial infection. Although our *in vitro* strain-competition data suggests that NPI is driving a host response rather than direct bacterial interaction. Biofilm formation is currently under investigation as a potential alternate mechanism for NPI prophylactic protection. Another area of future research and a caveat to this study is the limited information we have on the baseline microbiome of the murine prostate. While we demonstrate that no culturable bacteria are present in the uninfected prostate of NOD mice, it is possible that there is a microflora in the prostate that is only detectable by sequence analysis. In this circumstance it is possible that NPI and/or CP1 could exert their effects on host immune and tactile allodynia responses via modulation of this microflora. It is our contention however that this putative microflora would be relatively similar in abundance and diversity between mice at experimental outset as they are housed and caged together until 8 weeks of age and are purchased from the same vendor at the same time. These factors serve to optimize reproducibility between individual mice and experimental repeats.

We also hypothesize that NPI instillation primes the immune system of the urinary tract to react more effectively against invading pathogens such as CP1. In this context it is important to note that development of murine CP/CPPS is maintained long after the CP1 bacteria has been cleared^6^, indicating a role for loss of a balanced immune system that could be restored upon treatment with NPI. Indeed the expression of the chemokine and cytokine analytes increased with CP1-infection alone is blunted when NPI is given prophylactically. Pre-colonization by NPI followed by CP1-infection showed increased VEGF and IL4 expression when compared to CP1-alone (analysis 3) and decreased levels of CCL2/3 and 4, and IL17. When we compared analytes from mice with NPI then CP1 to NPI alone we observed increased expression of CCl4, CXCL1/2/9 and 10, and Lif, albeit at a much reduced level than that observed in mice infected with CP1-alone, demonstrating a blunted response that was incapable of inducing tactile allodynia. Notably absent from these increased analytes were cytokines such as IFNg, IL12b (P40 and P70), implicated in another model of CP/CPPS^30^, and IL17. VEGF expression was also shown to be increased in the NPI prophylactic group compared to either CP1 alone or NPI alone, which is interesting given that studies have shown that blockade of VEGF by an antibody reduced pelvic nociceptive responses in cyclophosphamide (CYP) cystitis^31^.

Taken together the data presented here represent a novel study on the role of both pathogenic and commensal bacterial strains in prostate health and disease. We focused on the ability of a human isolate from a healthy control, NPI, to prevent and reverse induction of tactile allodynia and inflammation by a virulent pathogen, CP1. Analyses of interplay between these two strains and their host reveal potential avenues for therapeutic intervention in patients with CP/CPPS and the contribution of the immune response to symptom development. Furthermore, it highlights a potential etiology for certain subcategories of prostatitis, whereby loss of a healthy prostate microflora could contribute to auto-immune activation and loss of tolerance initiated by pathogenic infection.

## Electronic supplementary material

Supplementary Figures
