# Supplementary material for: A prostate derived commensal Staphylococcus epidermidis strain prevents and ameliorates induction of chronic prostatitis by UPEC infection
Source: Sci Rep. 2018 Nov 27;8:17420. doi: 10.1038/s41598-018-35818-1 (PMC6258684; doi:10.1038/s41598-018-35818-1)

Supplementary Information:

Title: A prostate derived commensal *Staphylococcus epidermidis* strain prevents and ameliorates induction of chronic prostatitis by UPEC infection.

Authors: Stephen F. Murphy[1], Christel Hall[1], Joseph Done[1], Anthony J. Schaeffer[1] & Praveen Thumbikat[1*]

*Corresponding author

Email: thumbikat@northwestern.edu

Affiliation: [1] Dept. of Urology, Feinberg School of Medicine, Northwestern University, Chicago, IL 60611.

Supplementary Figure 1: Control plates for deferred growth inhibition assay. Plates depicted show a negative control "Blank" sample, and colonies grown individually as a single dot inoculum (upper plates) or as a spray/lawn (lower plates) alongside experimental groups.

Supplementary Figure 2: Comparison of prostate immune responses to CP1 infection versus NPI instillation. (A) Expression level of Luminex analytes in CP1 infected mice normalized to the NPI instilled group. Red bars denote increased expression >=1.4 fold, White, bars denote 1.4 to 0.8 fold (no change) and Green bars <= 0.8 fold decrease, normalized group depicted in Black. (B) STRING analyses of analytes with increased expression and (C) corresponding 5-most significantly represented GO terms. (D) STRING analyses of analytes with decreased expression and (E) corresponding 5-most significantly represented GO terms. N=1, with n=3/4 mice per group.

Supplementary Figure 3: Comparison of prostate immune responses to NPI treatment of CP1 infection versus CP1 alone. (A) Expression level of Luminex analytes in NPI treated-CP1 infected mice normalized to the CP1 infected group. Red bars denote increased expression >=1.4 fold, White, bars denote 1.4 to 0.8 fold (no change) and Green bars <= 0.8 fold decrease, normalized group depicted in Black. (B) STRING analyses of analytes with increased expression and (C) corresponding 5-most significantly represented GO terms. (D) STRING analyses of analytes with decreased expression and (E) corresponding 5-most significantly represented GO terms. N=1, with n=3/4 mice per group.

Supplementary Figure 4: Comparison of prostate immune responses to prophylactic treatment with NPI versus CP1 alone. (A) Expression level of Luminex analytes in NPI prophylaxis mice normalized to the CP1 infected group. Red bars denote increased expression >=1.4 fold, White, bars denote 1.4 to 0.8 fold (no change) and Green bars <= 0.8 fold decrease, normalized group depicted in

Black. (B) STRING analyses of analytes with increased expression and (C) corresponding 5-most significantly represented GO terms. (D) STRING analyses of analytes with decreased expression and (E) corresponding 5-most significantly represented GO terms. N=1, with n=3/4 mice per group.

Supplementary Figure 5: Comparison of prostate immune responses to prophylactic treatment with NPI versus NPI alone. (A) Expression level of Luminex analytes in NPI prophylaxis mice normalized to the NPI instilled group. Red bars denote increased expression >=1.4 fold, White, bars denote 1.4 to 0.8 fold (no change) and Green bars <= 0.8 fold decrease, normalized group depicted in Black. (B) STRING analyses of analytes with increased expression and (C) corresponding 5-most significantly represented GO terms. (D) STRING analyses of analytes with decreased expression and (E) corresponding 5-most significantly represented GO terms. N=1, with n=3/4 mice per group.

Supplementary Figure 6: Comparison of prostate immune responses to NPI treatment of CP1 infection versus NPI alone. (A) Expression level of Luminex analytes in NPI treated-CP1 infected mice normalized to the NPI instilled group. Red bars denote increased expression >=1.4 fold, White, bars denote 1.4 to 0.8 fold (no change) and Green bars <= 0.8 fold decrease, normalized group depicted in Black. (B) STRING analyses of analytes with increased expression and (C) corresponding 5-most significantly represented GO terms. (D) STRING analyses of analytes with decreased expression and (E) corresponding 5-most significantly represented GO terms. N=1, with n=3/4 mice per group.

Supplementary Figure 7: Immunological responses to CP1 infection, NPI instillation or combinations of both from iliac lymph nodes of treated mice. (A) Luminex chemokine and cytokine bead array data from lymph node tissues displayed as (i) heat-map of five comparisons between mouse groups. (ii) Tabular breakdown of comparisons performed. (B) (i-v) Individual

graphical representations of comparisons between analytes from each mouse group. Red bars denote increased expression >=1.4 fold, White, bars denote 1.4 to 0.8 fold (no change) and Green bars <= 0.8 fold decrease, normalized group depicted in Black. N=1, with n=1/2 mice per group.

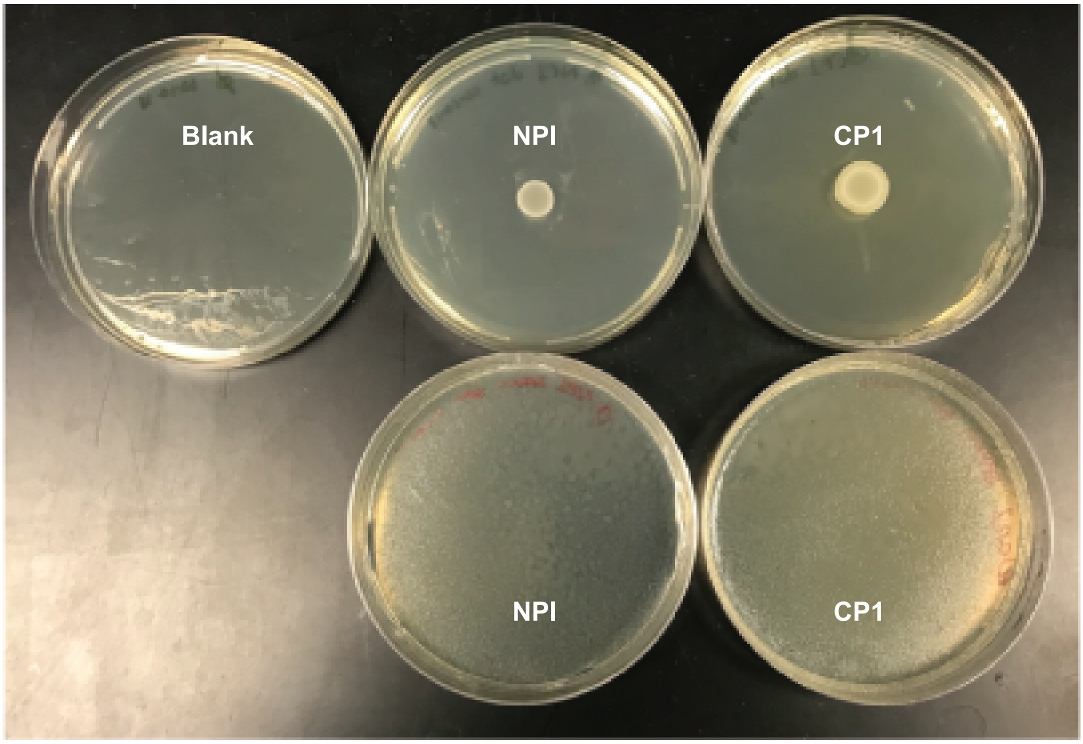
Blank
NPI
CP1
NPI
CP1

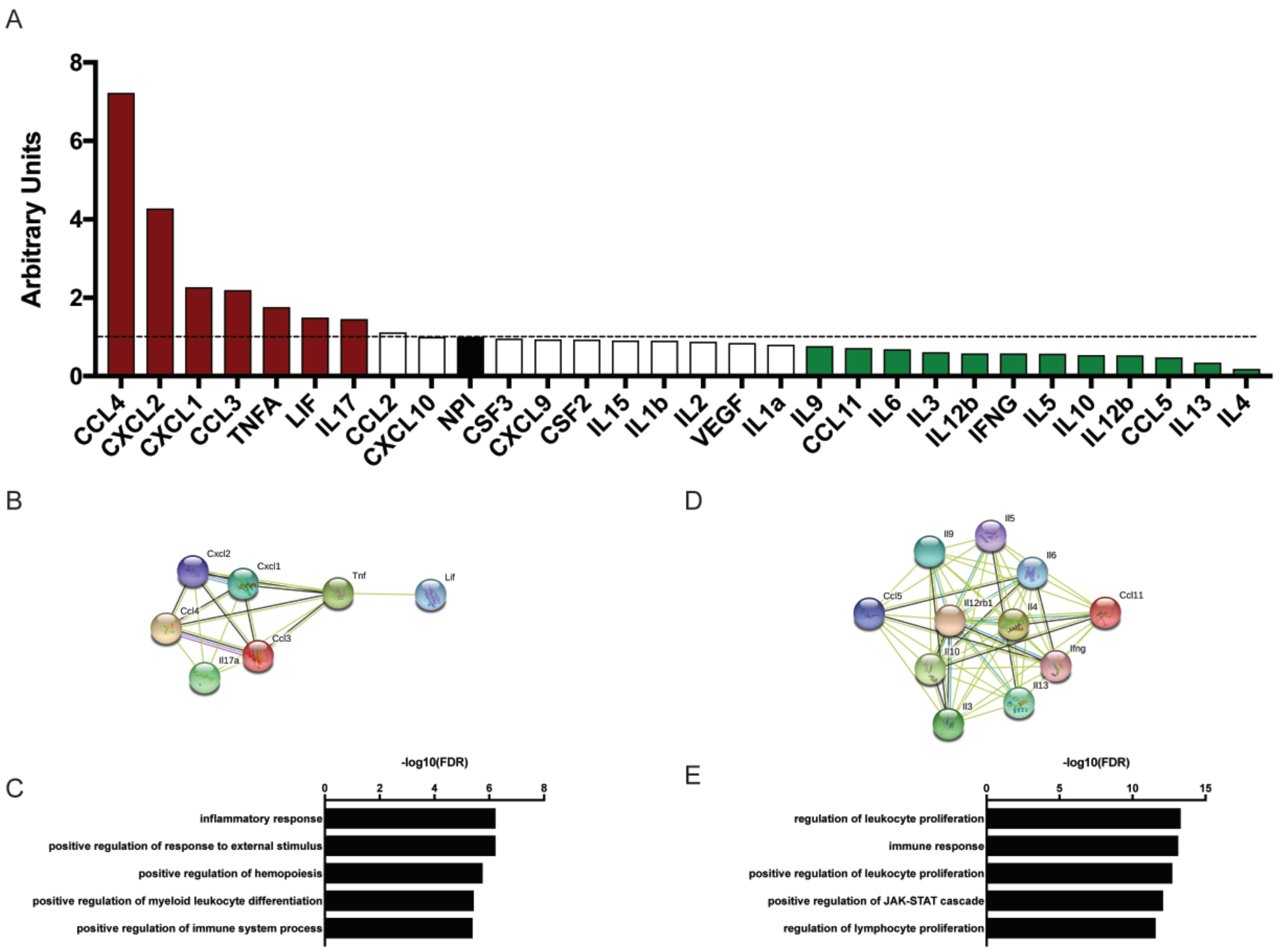
A
Arbitrary Units
8
6
4
2
0
CCL4
CXCL2
CXCL1
CCL3
TNFA
LIF
IL17
CCL2
CXCL10
NPI
CSF3
CXCL9
CSF2
IL15
IL1b
IL2
VEGF
IL1a
IL9
CCL11
IL6
IL3
IL12b
IFNG
IL5
IL10
IL12b
CCL5
IL13
IL4
B
Cxcl2
Cxcl1
Tnf
Lif
Ccl4
Ccl3
Il17a
D
Il5
Il9
Il6
Ccl5
Il12rb1
Il4
Ccl11
Il10
Ifng
Il13
Il3
C
-log10(FDR)
0
2
4
6
8
inflammatory response
positive regulation of response to external stimulus
positive regulation of hemopoiesis
positive regulation of myeloid leukocyte differentiation
positive regulation of immune system process
E
-log10(FDR)
0
5
10
15
regulation of leukocyte proliferation
immune response
positive regulation of leukocyte proliferation
positive regulation of JAK-STAT cascade
regulation of lymphocyte proliferation

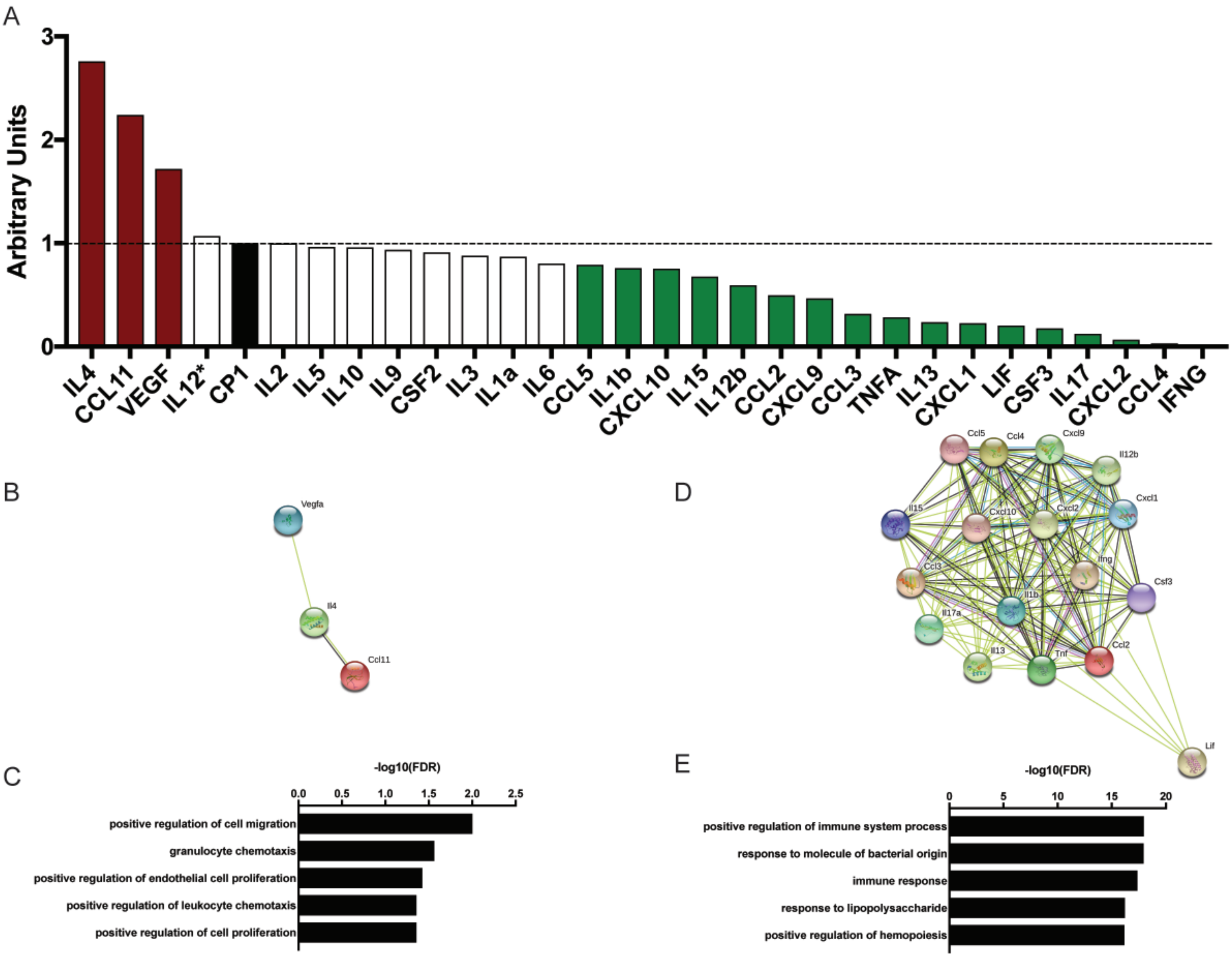

A
Arbitrary Units
3
2
1
0
IL4
CCL11
VEGF
IL12*
CP1
IL2
IL5
IL10
IL9
CSF2
IL3
IL1a
IL6
CCL5
IL1b
CXCL10
IL15
IL12b
CCL2
CXCL9
CCL3
TNFA
IL13
CXCL1
LIF
CSF3
IL17
CXCL2
CCL4
IFNG
B
Vegfa
Il4
Ccl11
C
-log10(FDR)
0.0
0.5
1.0
1.5
2.0
2.5
positive regulation of cell migration
granulocyte chemotaxis
positive regulation of endothelial cell proliferation
positive regulation of leukocyte chemotaxis
positive regulation of cell proliferation
D
Ccl5
Ccl4
Cxcl9
Il12b
Cxcl1
Il15
Cxcl10
Cxcl2
Ifng
Ccl3
Csf3
Il1b
Il17a
Ccl2
Il13
Tnf
Lif
E
-log10(FDR)
0
5
10
15
20
positive regulation of immune system process
response to molecule of bacterial origin
immune response
response to lipopolysaccharide
positive regulation of hemopoiesis

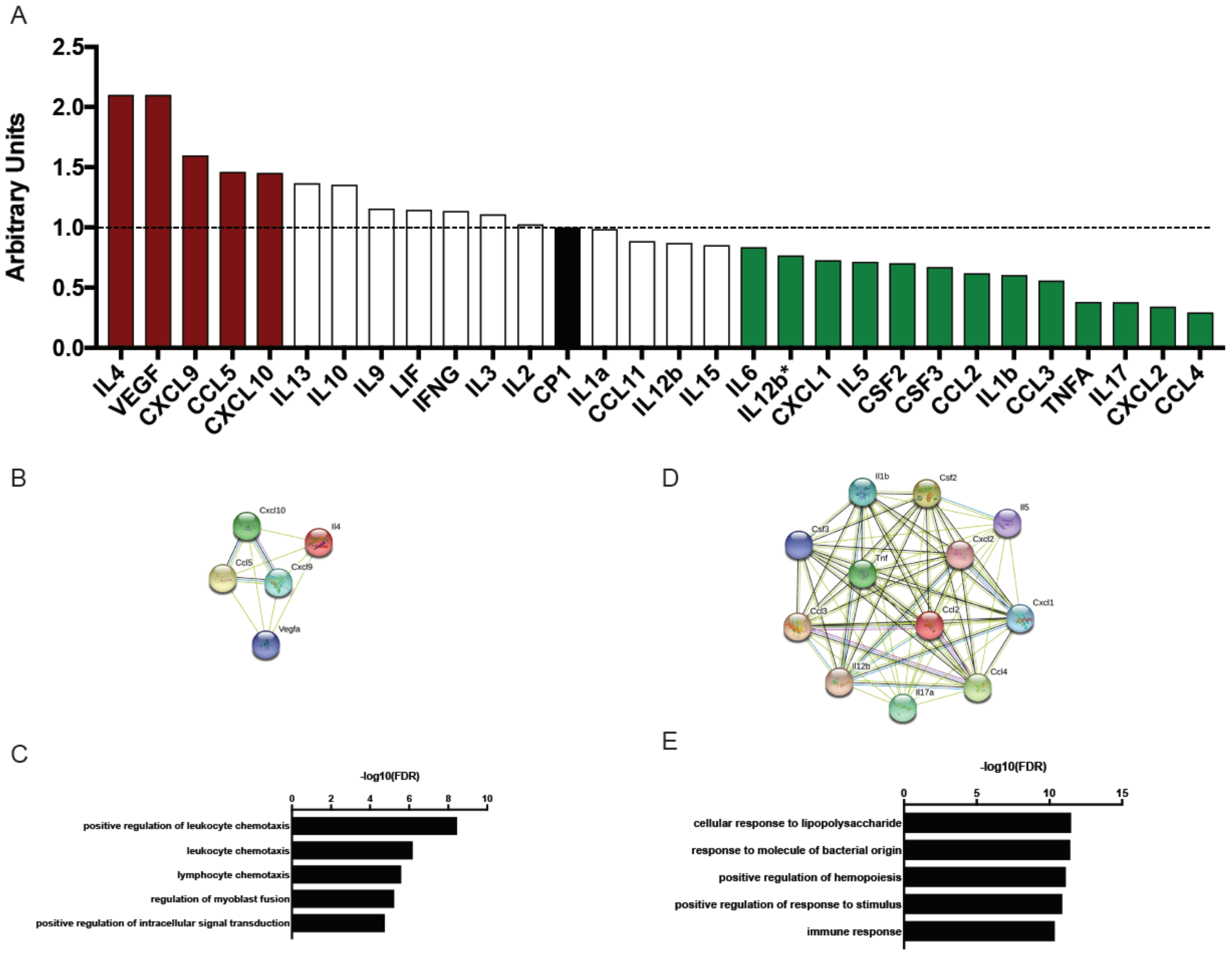
A
Arbitrary Units
2.5
2.0
1.5
1.0
0.5
0.0
IL4
VEGF
CXCL9
CCL5
CXCL10
IL13
IL10
IL9
LIF
IFNG
IL3
IL2
CP1
IL1a
CCL11
IL12b
IL15
IL6
IL12b*
CXCL1
IL5
CSF2
CSF3
CCL2
IL1b
CCL3
TNFA
IL17
CXCL2
CCL4
B
Cxcl10
Il4
Ccl5
Cxcl9
Vegfa
D
Il1b
Csf2
Il5
Csf3
Cxcl2
Tnf
Cxcl1
Ccl3
Ccl2
Il12b
Ccl4
Il17a
C
-log10(FDR)
0
2
4
6
8
10
positive regulation of leukocyte chemotaxis
leukocyte chemotaxis
lymphocyte chemotaxis
regulation of myoblast fusion
positive regulation of intracellular signal transduction
E
-log10(FDR)
0
5
10
15
cellular response to lipopolysaccharide
response to molecule of bacterial origin
positive regulation of hemopoiesis
positive regulation of response to stimulus
immune response

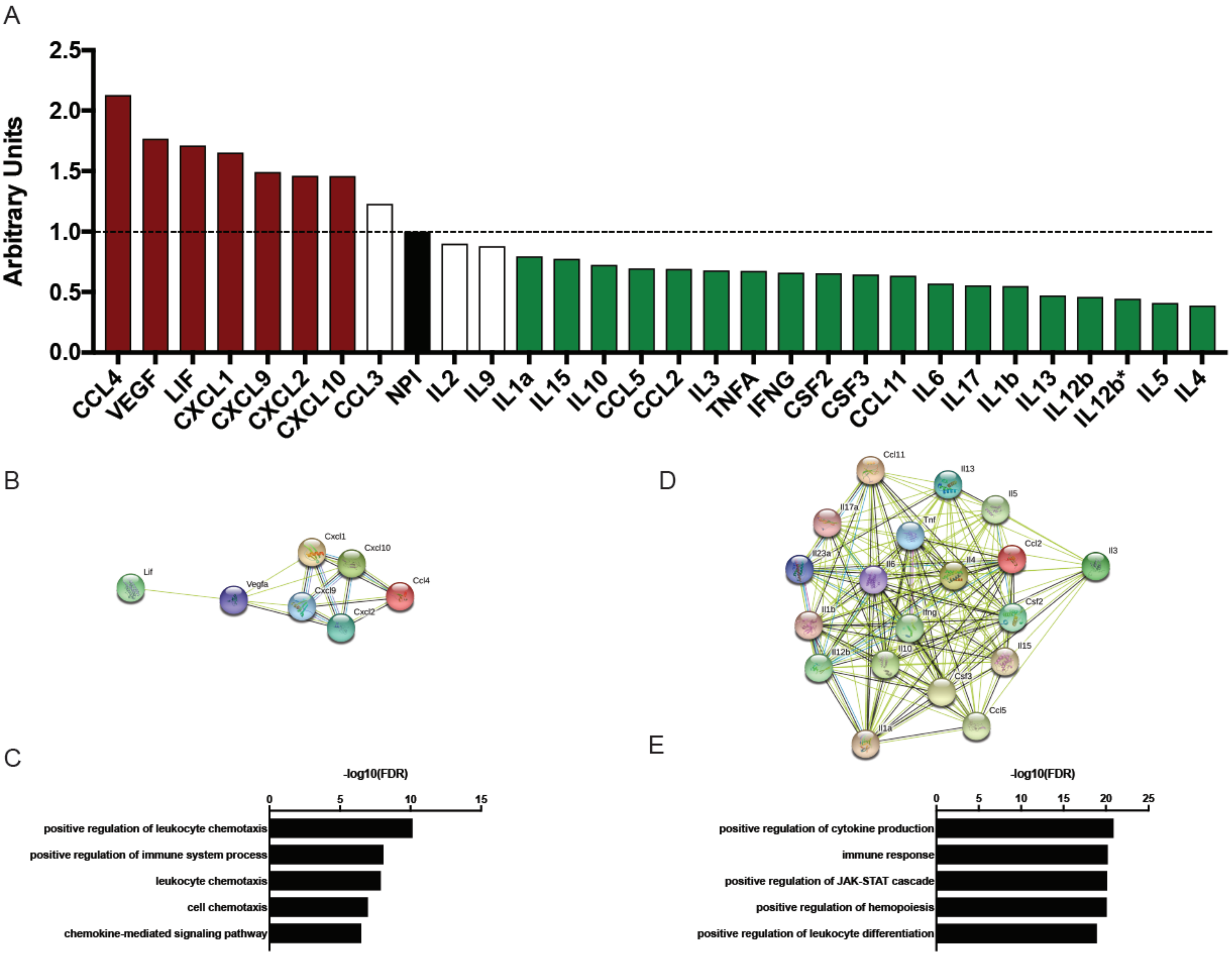

A
Arbitrary Units
0.0
0.5
1.0
1.5
2.0
2.5
CCL4
VEGF
LIF
CXCL1
CXCL9
CXCL2
CXCL10
CCL3
NPI
IL2
IL9
IL1a
IL15
IL10
CCL5
CCL2
IL3
TNFA
IFNG
CSF2
CSF3
CCL11
IL6
IL17
IL1b
IL13
IL12b
IL12b*
IL5
IL4
B
Lif
Vegfa
Cxcl1
Cxcl10
Ccl4
Cxcl9
Cxcl2
D
Ccl11
Il13
Il5
Il17a
Tnf
Ccl2
Il3
Il23a
Il6
Il4
Il1b
Ifng
Csf2
Il12b
Il10
Il15
Csf3
Ccl5
Il1a
C
-log10(FDR)
0
5
10
15
positive regulation of leukocyte chemotaxis
positive regulation of immune system process
leukocyte chemotaxis
cell chemotaxis
chemokine-mediated signaling pathway
E
-log10(FDR)
0
5
10
15
20
25
positive regulation of cytokine production
immune response
positive regulation of JAK-STAT cascade
positive regulation of hemopoiesis
positive regulation of leukocyte differentiation

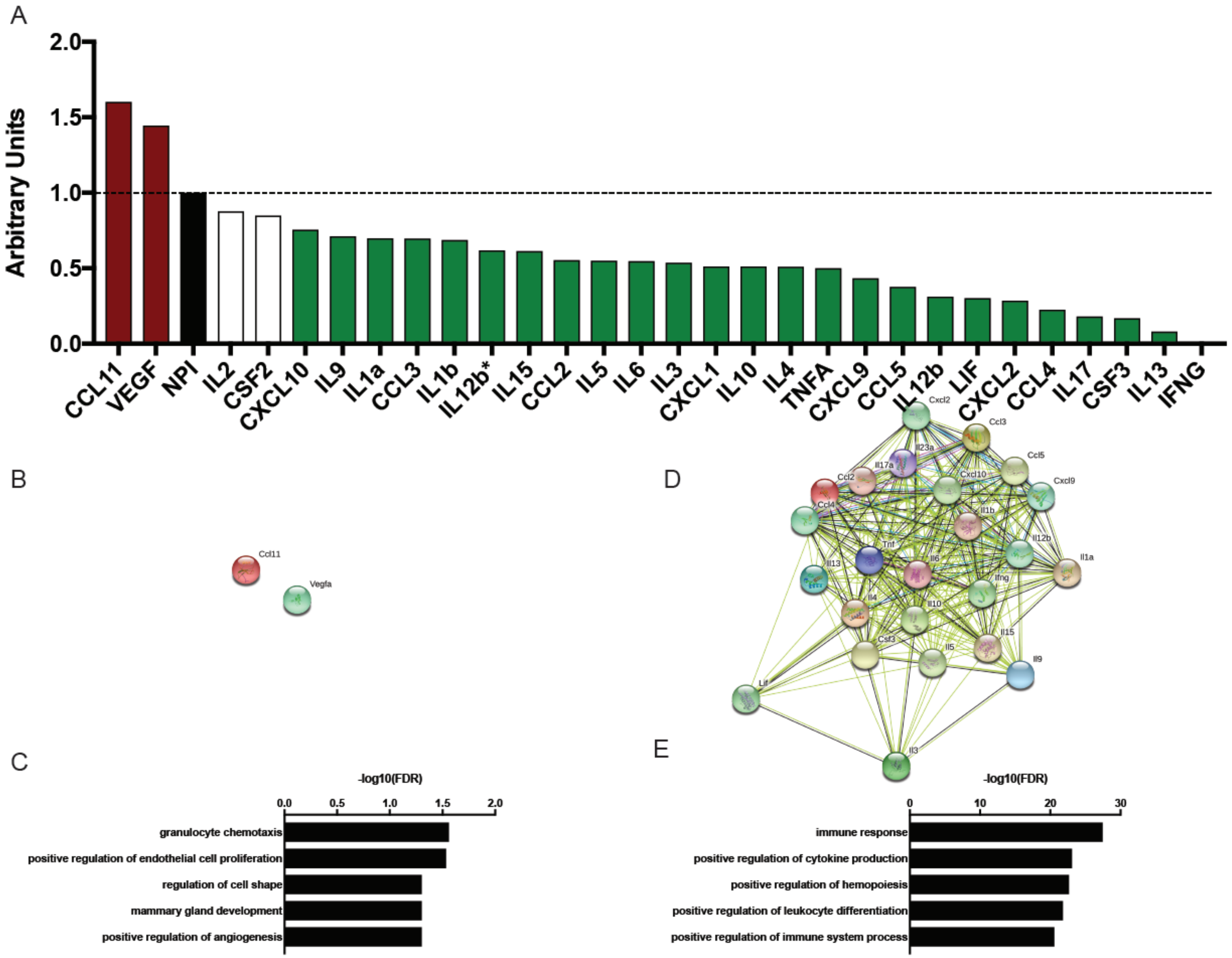
A
Arbitrary Units
2.0
1.5
1.0
0.5
0.0
CCL11
VEGF
NPI
IL2
CSF2
CXCL10
IL9
IL1a
CCL3
IL1b
IL12b*
IL15
CCL2
IL5
IL6
IL3
CXCL1
IL10
IL4
TNFA
CXCL9
CCL5
IL12b
LIF
CXCL2
CCL4
IL17
CSF3
IL13
IFNG
B
Ccl11
Vegfa
D
Cxcl2
Ccl3
Il23a
Ccl5
Il17a
Ccl2
Cxcl10
Cxcl9
Ccl4
Il1b
Il12b
Tnf
Il1a
Il13
Il6
Ifng
Il4
Il10
Csf3
Il15
Il5
Il9
Lif
Il3
C
-log10(FDR)
0.0
0.5
1.0
1.5
2.0
granulocyte chemotaxis
positive regulation of endothelial cell proliferation
regulation of cell shape
mammary gland development
positive regulation of angiogenesis
E
-log10(FDR)
0
10
20
30
immune response
positive regulation of cytokine production
positive regulation of hemopoiesis
positive regulation of leukocyte differentiation
positive regulation of immune system process

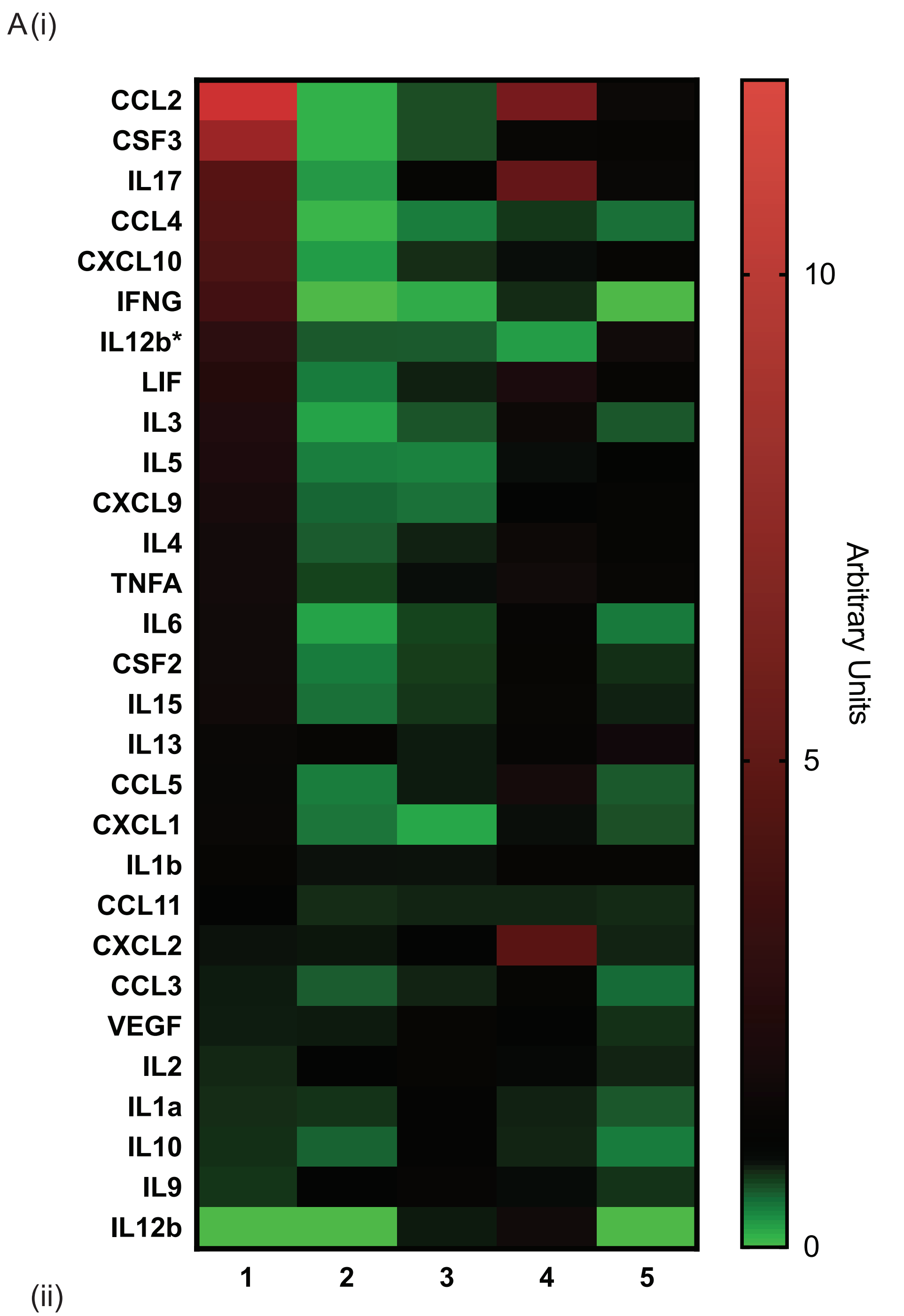

(ii)

Comparison	Treatment Group	Control Group	Immune Response to:
1	CP1	NPI	Induction of Tactile Allodynia
2	CP1 then NPI	CP1	Resolution of Tactile Allodynia by NPI
3	NPI then CP1	CP1	Prevention of Tactile Allodynia by NPI
4	NPI then CP1	NPI	Reduced Tactile Allodynia Induction by CP1
5	CP1 then NPI	NPI	Resolved response to CP1

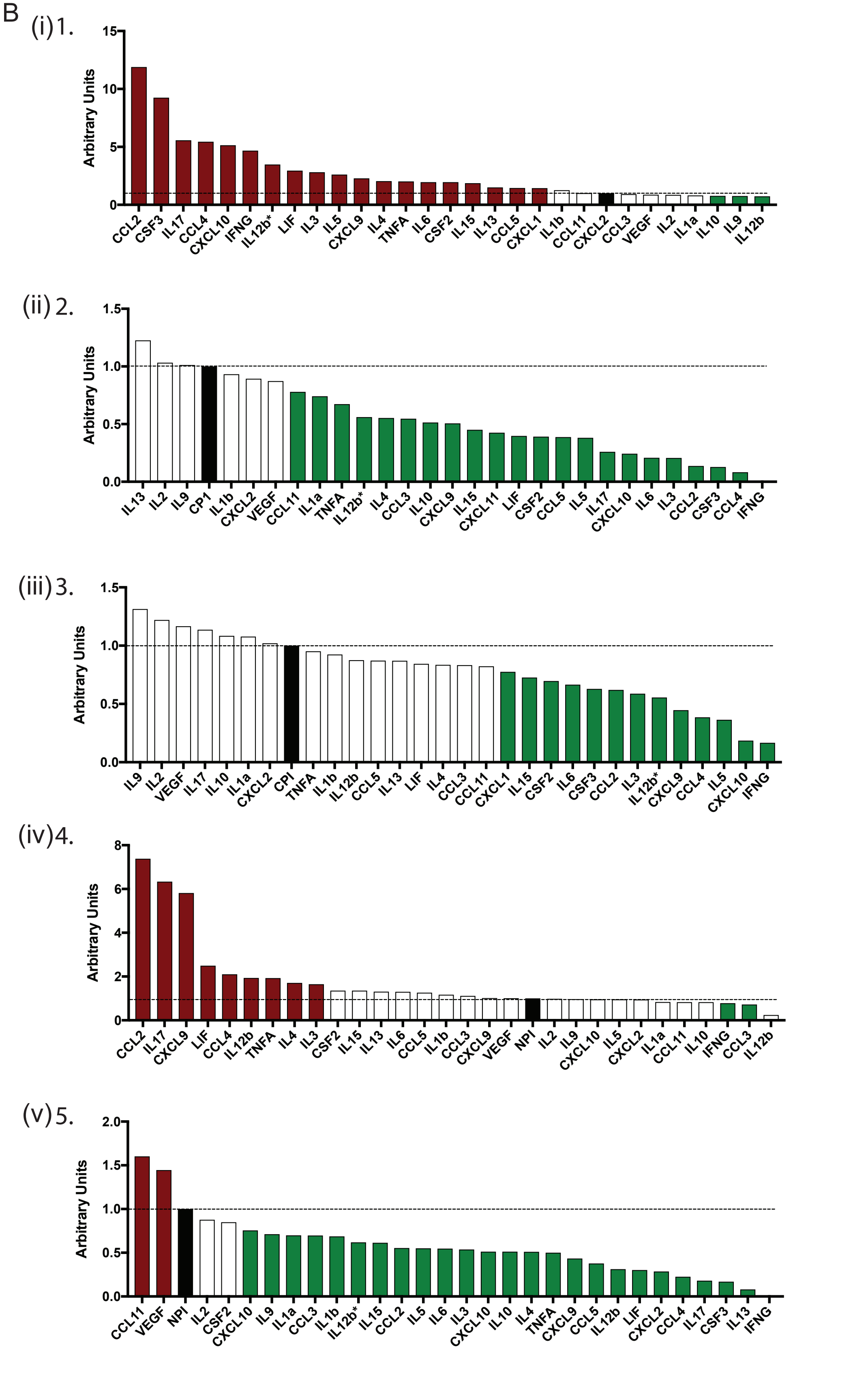

Supplement: Supplementary file 1 — Supplementary Figures [file 41598_2018_35818_MOESM1_ESM.pdf]
